# Transcriptome Analysis of Different Chinese Cabbage Varieties Under Cd and Pb Stresses

**DOI:** 10.3390/ijms26188945

**Published:** 2025-09-14

**Authors:** Shiqi Peng, Hao Zhang, Junlei Wang, Liyuan Mu, Sijing Sun, Ao Li, Naiming Zhang, Li Bao

**Affiliations:** 1 College of Resources and Environment, Yunnan Agricultural University, Kunming 650201, China; 13529637422@163.com (S.P.); zhanghao74586@163.com (H.Z.); 18131119221@163.com (J.W.); 18895890735@163.com (L.M.); sunsijwxsa@163.com (S.S.); 13759478903@163.com (A.L.); 18132676221@163.com (N.Z.); 2 Yunnan Laboratory of Improvement of Soil Fertility and Pollution Remediation, Kunming 650201, China

**Keywords:** heavy metal stress, transcriptome sequencing, molecular response, crop growth

## Abstract

In recent years, soil heavy metal pollution has become increasingly serious, particularly Cd and Pb pollution, and heavy metals have been accumulating in soil–crop systems, posing great risks to human health. In this study, four Chinese cabbage varieties with different Cd and Pb accumulation traits were cultivated using hydroponics and transcriptome sequencing technology to reveal the response mechanism of Chinese cabbage to Cd and Pb stress at the molecular level. The numbers of upregulated genes in Harmony Express (H) and Ziwei F1 (F) were 2904 and 3004, respectively, under Cd stress (0 mg/L vs. 80 mg/L), whereas the numbers of upregulated genes in Green Crown (L) and Suzhou Green (S) were 3424 and 2724, respectively, under Pb stress (0 mg/L vs. 1200 mg/L). GO enrichment analysis revealed that 52 functional subgroups were enriched in H0 vs. H80 and F0 vs. F80, and 79 functional subgroups were enriched in L0 vs. L1200 and S0 vs. S1200. KEGG enrichment indicated that secondary metabolite biosynthesis, metabolic pathways, and phenylpropanoid biosynthesis are important regulators of the response to Cd and Pb stress in Chinese cabbage. In addition, genes related to antioxidant enzymes (e.g., CAT and glutathione transferase), metal transporter proteins (e.g., ABC), mitogen-activated protein kinases, and calmodulin were significantly differentially expressed, suggesting that they are jointly involved in the detoxification of Chinese cabbage in response to heavy metal stress. In total, 881 and 858 differentially expressed genes (DEGs) in the transcription factor family responded to Cd and Pb stress, respectively. This study reveals the response mechanism of Chinese cabbage to Cd and Pb stress at the molecular level and provides a theoretical basis for the cultivation of low-Cd and low-Pb-enriched varieties and the mining of heavy metal tolerance genes.

## 1. Introduction

Soil systems are an important part of ecosystems, and in recent years, with rapid socioeconomic development, land-use patterns have undergone substantial change in a short period. Heavy metal contamination of soils due to disturbances in the geological background as well as anthropogenic activities has become a major environmental issue of wide concern on a global scale [1,2,3]. Heavy metals in soil are highly toxic, persistent, and bioaccumulative [4]. Heavy metals can disrupt soil structure and affect the soil-crop system, leading to decreased yield and quality of agricultural products [5]. The bioaccumulative properties of soil heavy metals typically enrich them in agricultural products through the soil-crop system, and they then enter the human body through the food chain; long-term exposure to and intake of heavy metals can pose great risks to human health [6]. Leafy vegetables have high capacity for heavy metals [7]. Among these, Cd is one of the main elements enriched in vegetables, and long-term exposure to Cd can lead to cancer, inhibit protein synthesis, and trigger fractures [8]. Conversely, low concentrations of Pb have a certain promotional effect on seed germination and plant growth and development, whereas excessive Pb in the environment may lead to its enrichment in crops, interfering with the normal physiological and biochemical processes of plants and seriously affecting their normal growth [9]. Excessive Pb can cause kidney damage in humans, affect the reproductive system, cause miscarriages, and impede intellectual development in children [10].

When plants are subjected to stress, they activate resistance genes in the body and then activate transcription factors to respond to biotic or abiotic stresses and form various types of phytohormones and functional proteins to participate in the complex resistance process [11]. Taking heavy metal stress as an example, metal ions enter plant cells and induce differential gene expression through signaling mechanisms, thereby activating cellular responses. The regulatory mechanisms involved in plant responses to heavy metal stress mainly include signal sensing, regulation of related transcription factors, activation of transporter proteins and antioxidant enzymes, and the synthesis of biochelates [12]. At the cellular and molecular levels, heavy metals have various toxic effects on plants, interfering with important processes, such as gene expression and protein synthesis, thereby negatively affecting plant growth and development. During long-term adaptation to the environment, plants develop regulatory and defense mechanisms for various adverse conditions [13]. Because the toxic effects of heavy metals on plants are multifaceted, the detoxification mechanisms of plants against heavy metals are the combined effects of various physiological processes. Plants detoxify heavy metals mainly through antioxidant defense systems, chelation and compartmentalization [14]. Transcriptome sequencing (RNA sequencing [RNA-Seq]) is a recently developed technology that utilizes high-throughput sequencing to sequence all cDNAs in a given specimen. Using transcriptome sequencing, we can quickly and accurately understand plant gene expression at the molecular level during stress states [15].

Most studies on heavy metal enrichment in crops under heavy metal stress have focused on model plants (rice, *Arabidopsis thaliana*) and hyper-enriched plants (Southeast *Sedum* and *Helianthus annuus*) [16], whereas studies on the effects of stress on leafy vegetables, particularly those under different levels of stress, are scarce. Therefore, in the present study, four Cd- and Pb-accumulating Chinese cabbage varieties with different cumulative traits (Harmony Express (H) for low Cd enrichment, Ziwei F1 (F) for high Cd enrichment, Green Crown (L) for low Pb enrichment, and Suzhou Green (S) for high Pb enrichment) were cultivated using hydroponics. Root samples were collected to detect DEGs using transcriptome sequencing to explore the functional annotation, classification, and metabolic enrichment pathways of the DEGs. Simultaneously, the genes and transcription factors related to the detoxification mechanism under heavy metal stress were localized: for example, H showed significant upregulation of genes related to the activity of related antioxidant enzymes and metal transport, and L showed greater induction of genes related to metal transport, signal perception, and transduction, in contrast to S. To analyze the molecular mechanism of heavy metal stress in Chinese cabbage, varieties with different cumulative traits were grown and studied to provide a theoretical basis for the cultivation of low-enriched leafy vegetable varieties and the study of tolerance genes. Given the differences in Cd and Pb accumulation and among different varieties of Chinese cabbage, the production of safe agricultural products on heavy metal-contaminated farmland has broad application prospects. Rational production planning is of great significance for ensuring food safety.

## 2. Results

### 2.1. Effects of Cd and Pb Stress on the Growth Indices of Chinese Cabbage

**Figure 1:**
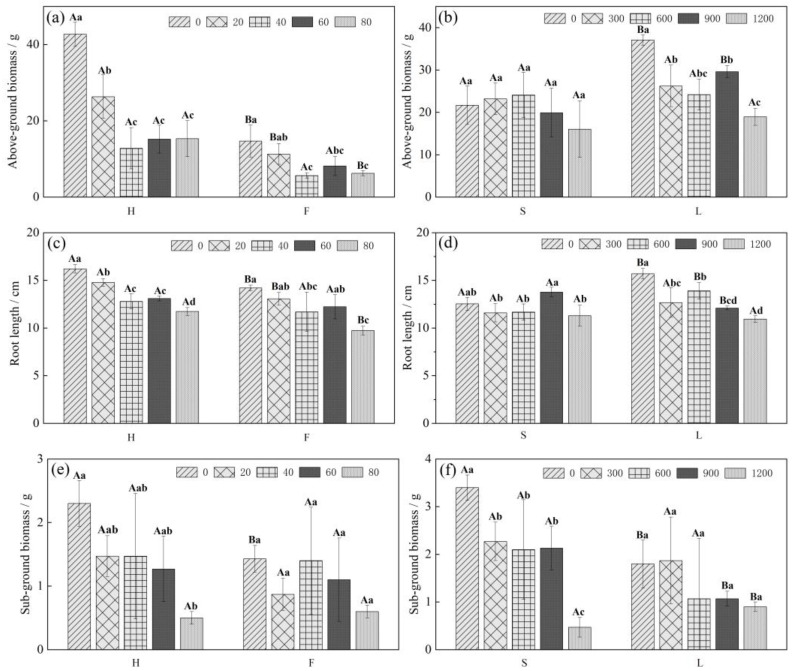
Effect of Cd and Pb stress on growth indices of Chinese cabbage ((**a**), (**c**), and (**e**) are Cd treatments; (**b**), (**d**), and (**f**) are Pb treatments). Note: Different lowercase letters indicate that the difference between different treatments of the same variety is significant (*p* < 0.05); different uppercase letters indicate that the difference between different varieties under the same treatment is significant (*p* < 0.05).

As shown in Figure 1, different Cd and Pb concentrations affected the biomass and root length of the Chinese cabbage varieties to different degrees. Under Cd stress, the aboveground/underground biomass and root length of varieties H and F decreased with increasing Cd concentrations. At a Cd concentration of 40 mg/L, the aboveground biomass of the H and F varieties decreased by 70.05% and 61.45% compared with that of H0 and F0, respectively, whereas at a Cd concentration of 80 mg/L, the root lengths of the H and F varieties decreased by 27.57% and 31.46% compared with those of H0 and F0, respectively, and underground biomass decreased by 78.26% and 58.14%, respectively. The differences in growth indicators under varying concentrations showed differing levels of significance, indicating that high-enrichment and low-enrichment Chinese cabbage varieties exhibit distinct sensitivities to Cd stress at different concentrations. The aboveground/underground biomass and root length of Chinese cabbage varied with increasing Pb concentrations under different Pb treatments. All three growth indices of both varieties were the lowest at a Pb concentration of 1200 mg/L, and the aboveground biomass of the L and S varieties decreased by 48.92% and 26.11%, the underground biomass by 50.00% and 86.27%, and the root length by 30.36% and 9.84%, respectively, when compared with the 0 mg/L treatment.

### 2.2. Cd and Pb Accumulation in Different Chinese Cabbage Varieties

The Cd absorption and accumulation capacities of the different Chinese cabbage varieties exhibited significant differences (Table 1). In this study, for the aboveground part of Chinese cabbage, the variety with the highest Cd content was F (10.60 mg/kg), and that with the lowest Cd content was H (4.72 mg/kg); the Cd content of F was 2.25 times higher than that of H. The variety with the highest Pb content was S (18.00 mg/kg), and the lowest was L (1.80 mg/kg); the Pb content of S was 10 times that of L. Subground, the variety with the highest Cd content was F (17.69 mg/kg), and that with the highest Pb content was S (245.32 mg/kg), respectively.

### 2.3. Cd and Pb Bioconcentration Factor (BCF) and Transfer Factor (TF) of Different Varieties of Chinese Cabbage

The BCF is an indicator of a plant’s ability to enrich heavy metals, and the TF is an indicator of the ability of heavy metal transfer and distribution in the plant; the larger the BCF value, the stronger the plant’s ability to absorb enriched heavy metals, and the larger the TF, the stronger the plant’s ability to transfer heavy metals. In this study, the BCF and TF values for the four different varieties of Chinese cabbage were calculated (Table 2); the BCF value of F for Cd was the largest at 2.02, the BCF value of H for Cd was the smallest at 0.90, and the TF value of H for Cd was the smallest at 0.50. The BCF value of S for Pb was the highest (0.059), the BCF value of L for Pb was the lowest (0.006), and the TF value of L for Pb was the lowest (0.013).

### 2.4. Quality Assessment of Sequencing Results and Statistics for DEGs

Twenty-four samples with three biological replicates each were subjected to transcriptome analysis in the control (F0, H0) and treatment (F80, H80) groups of Chinese cabbage with high and low accumulation under Cd stress, and in the control (S0, L0) and treatment (S1200, L1200) groups of Chinese cabbage with high and low accumulation under Pb stress. Their sequencing results are shown in Table A1 and Table A2. In the Cd and Pb treatment tests, the Q20 values were all >97%, the GC contents were all >46%, and the sequencing results were of good quality and consistent with the subsequent comparative transcriptome analysis [17]. Using the genome of *Brassica campestris* L. ssp. (*Brassica rapa* L.), a variety of Chinese cabbage subspecies with flower shoots, as the reference genome for the comparison of sequenced fragments, the comparison and unique comparison rates of Cd treatment were 84.53–86.20% and 80.21–83.47%, respectively, whereas the comparison rate of Pb treatment was 79.12–86.09%, the unique comparison rate was 77.09%, and the unique matching rate ranged from 77.03% to 83.43%. The degree of matching between sequenced fragments and reference genes was high, and the sequencing results could be used for the subsequent analysis of DEGs to ensure the accuracy and reliability of the results [18].

**Figure 2:**
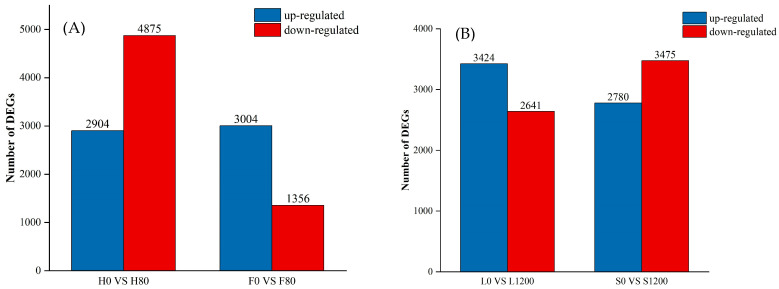
Statistics for DEGs in each variety under Cd and Pb stress. ((**A**) is Cd treatment, (**B**) is Pb treatment.)

Based on the results of differential analysis, genes with FDR < 0.05 and |log2FC| > 2 were selected as significant differential genes in this study to ensure the reliability of the results. As shown in Figure 2, in H0 vs. H80, F0 vs. F80, L0 vs. L1200, and S0 vs. S1200, the data for DEGs in the H varieties were significantly higher than those in the high cumulative F varieties under Cd stress, whereas the difference in the number of genes between the L and S varieties was not significant under Pb stress. These results provided a database for the subsequent analysis of DEGs, which in turn provided clues for the study of gene function and changes in biological pathways.

### 2.5. Gene Ontology (GO) Enrichment Analysis of DEGs

#### 2.5.1. GO Enrichment Analysis of DEGs Under Cd Stress

**Figure 3:**
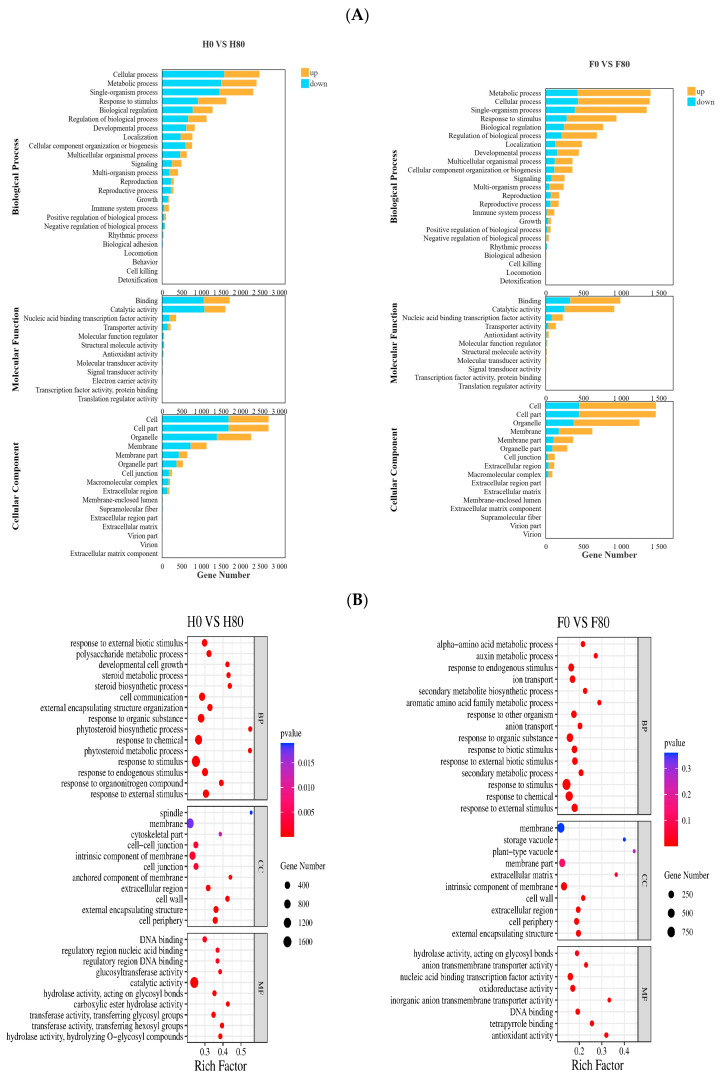
GO classification of differential genes and GO enrichment analysis of differentially expressed genes under Cd stress. (**A**) is the stacked bar chart of genes with upregulation and downregulation across three categories the number and proportion of genes upgraded and downgraded in the three categories; (**B**) is GO enrichment bubble chart.

The screened DEGs were categorized using GO, which has three ontologies describing the biological process (BP), molecular function (MF), and cellular component (CC) of the genes. As shown in Figure 3A, the DEGs of varieties in H0 vs. H80 contained 24, 12, and 16 functional groups, respectively; in F0 vs. F80, there were 23, 11, and 16 functional groups, and the BP types had the highest proportion of cellular and metabolic processes, whereas MF and CC types had the highest proportions of binding and cells, respectively.

The DEGs of Chinese cabbage under Cd stress were analyzed using GO enrichment, as shown in Figure 3B. The DEGs of both H and F varieties in the BP category were mainly enriched in response to external stimuli, response to stimuli, and response to chemicals; those in the CC category were cell periphery, external encapsulating structure, and extracellular region. In the enrichment of MF categories, the H and F varieties were different: H varieties were different in hydrolase activity, hydrolyzing O-glycosyl compounds, transfer activity, transferring hexosyl groups, transfer activity, transferring glycosyl groups, carboxylic acid ester hydrolase activity, and other BPs. DEGs in F varieties were mainly enriched in BPs, such as anion transmembrane transporter activity, inorganic anion transmembrane transporter activity, and substrate-specific transmembrane transporter activity.

#### 2.5.2. GO Enrichment Analysis of DEGs Under Pb Stress

**Figure 4:**
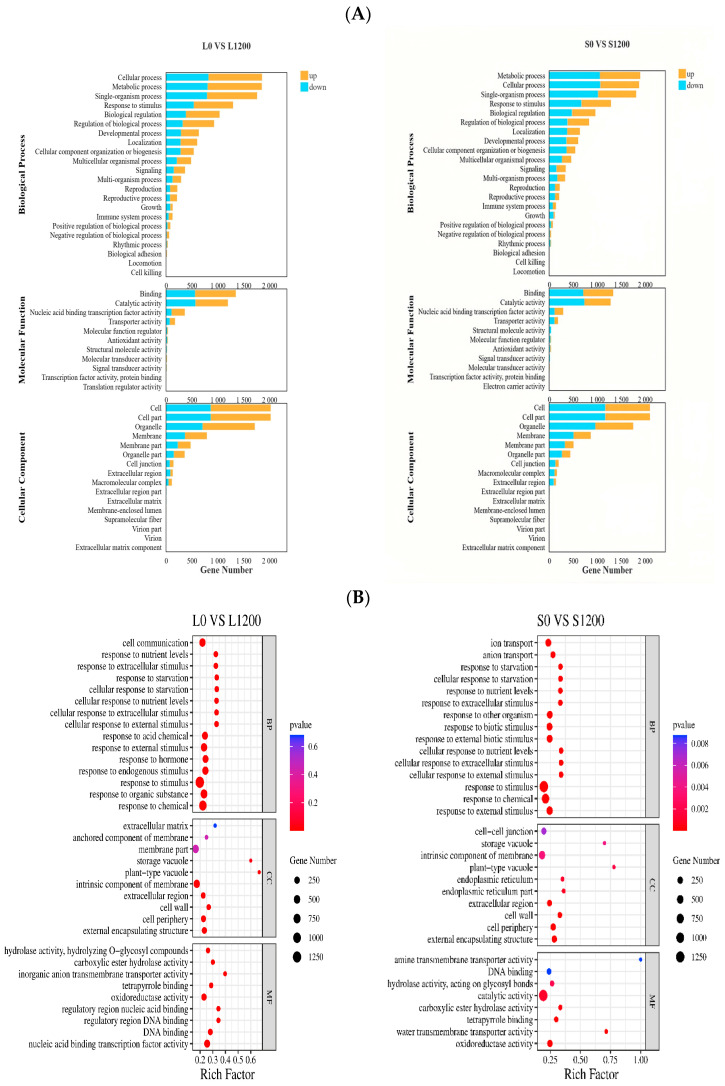
GO classification of differential genes and GO enrichment analysis of differentially expressed genes under Pb stress. (**A**) is the stacked bar chart of genes with upregulation and downregulation across three categories the number and proportion of genes upgraded and downgraded in the three categories; (**B**) is GO enrichment bubble chart.

As shown in Figure 4A, under Pb stress, the number of upregulated DEGs was higher than that of downregulated genes in L0 vs. L1200, whereas the number of downregulated genes was higher than the number of upregulated genes in S0 vs. S1200. The BP type had the highest proportion of cellular processes, metabolic processes, single-organism processes, responses to stimuli, and biological regulation; the MF type had the highest proportion of binding and catalytic activity; and the CC type had the highest proportion of cells and cells, followed by organelles.

GO enrichment analysis of the DEGs under Pb stress in *Chinese cabbage* was conducted. As shown in Figure 4B, the DEGs of L and S under Pb stress were heavily enriched in the entries related to the stress response in BPs, such as response to chemicals, response to organic substances, response to stimuli, and response to stimuli in the BP category.

### 2.6. Kyoto Encyclopedia of Genes and Genomes (KEGG) Enrichment Analysis of DEGs

**Figure 5:**
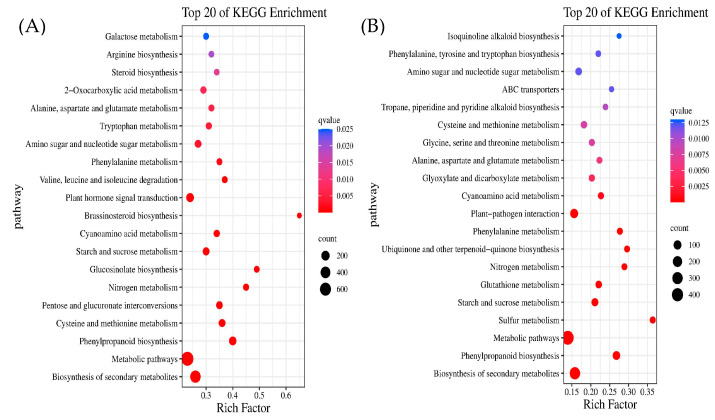

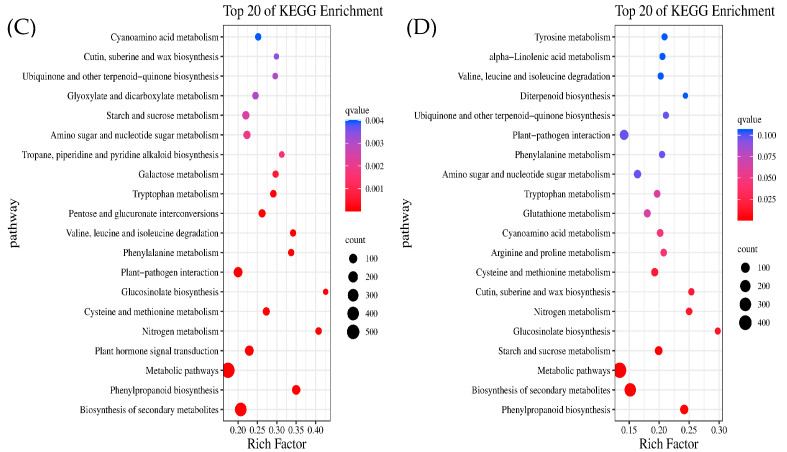
KEGG enrichment analysis of DEGs under Cd and Pb stress ((**A**) is H0 vs. H80, (**B**) is F0 vs. F80, (**C**) is L0 vs. L1200, (**D**) is S0 vs. S1200).

KEGG pathways enriched in DEGs of H0 vs. H80 and F0 vs. F80 under Cd stress were analyzed, and 2533 and 1580 genes were significantly enriched in H0 vs. H80 and F0 vs. F80, respectively; KEGG-enriched bubble diagrams were plotted by selecting the top 20 pathways that were significantly enriched. As shown in Figure 5A,B, the most highly enriched metabolic pathways in both genotypes of Chinese cabbage were biosynthesis of secondary metabolites, metabolic pathways, and phenylpropanoids, and the pathways specifically enriched for species F were sulfur metabolism, ubiquinone and other terpenoid-quinone biosynthesis, glycine, serine, and threonine metabolism, and the metabolism of secondary metabolites, metabolic pathways, phenylpropanoid biosynthesis, serine and threonine metabolism, and ABC transporters.

Under Pb stress, 2052 and 2057 genes in L0 vs. L1200 and S0 vs. S1200 were significantly enriched in 29 and 27 pathways, respectively, and the top 20 pathways were selected to plot KEGG enrichment bubbles. As shown in Figure 5C,D, the most highly enriched metabolic pathways in both the L and S genotypes of Chinese cabbage were phenylpropanoid biosynthesis, biosynthesis of secondary metabolites, and metabolic pathways, with the same enrichment in the first 10 pathways, including nitrogen metabolism, glucosinolate biosynthesis, and leucine and isoleucine degradation.

### 2.7. Analysis of Genes Related to Cd and Pb Stress Defense and Detoxification

#### 2.7.1. DEGs Related to Reactive Oxygen Species Scavenging System

**Figure 6:**
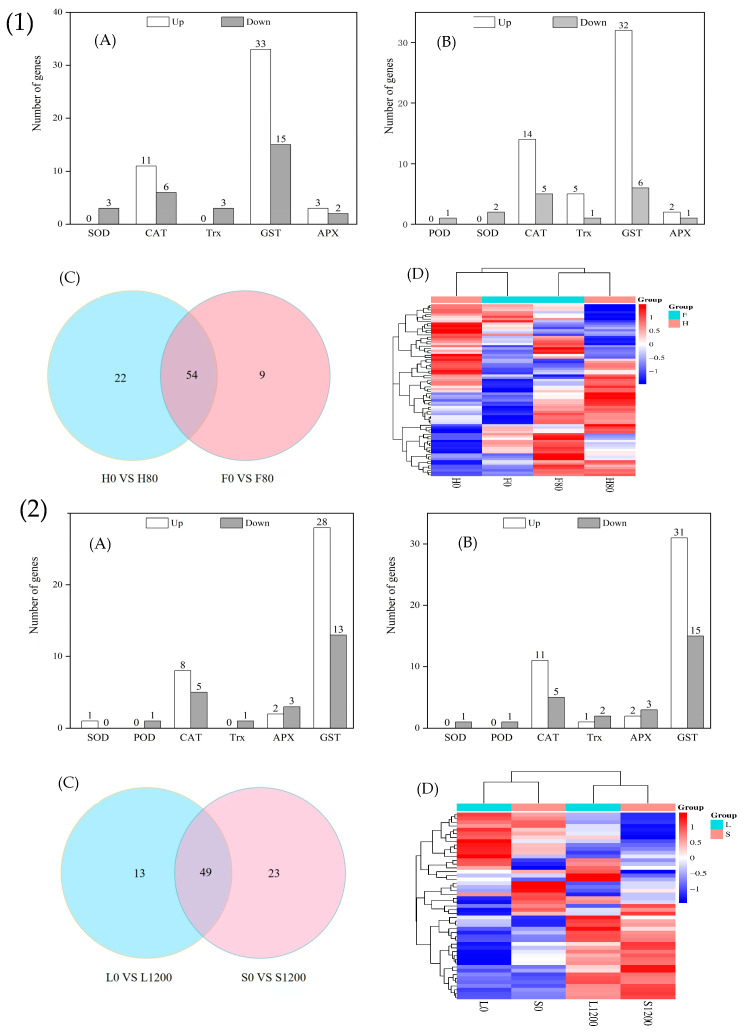
Number of significantly different genes associated with reactive oxygen species scavenging in H and F varieties under Cd stress, and L and S varieties under Pb stress. (**1**)(**A**) is number of significantly different genes associated with reactive oxygen species scavenging in H under Cd stress; (**1**)(**B**) is number of significantly different genes associated with reactive oxygen species scavenging in F under Cd stress; (**1**)(**C**) is wayne plots; (**1**)(**D**) is clustered heatmaps. (**2**)(**A**) is number of significantly different genes associated with reactive oxygen species scavenging in L under Pb stress; (**2**)(**B**) is number of significantly different genes associated with reactive oxygen species scavenging in S under Pb stress; (**2**)(**C**) is wayne plots; (**2**)(**D**) is clustered heatmaps. Note: In a Wayne diagram, the overlapping numbers represent the number of shared DEGs for different varieties, while the non-overlapping numbers represent the number of DEGs for a specific variety, as above below.

The DEGs related to the reactive oxygen species scavenging system in the H and F varieties of Chinese cabbage under Cd stress are shown in Figure 6. A total of 76 and 69 DEGs were significantly expressed in the H and F varieties after Cd stress, respectively, as shown in the Wayne plots and clustered heatmaps (Figure 6(1)C,D). A total of 54 DEGs associated with the reactive oxygen species scavenging system in the H and F varieties were significantly expressed in both varieties. Twelve genes coded for catalase (CAT) and 37 genes for glutathione S-transferase (GST), each of which had genes with opposite upregulation and downregulation in the H and F varieties. This indicates that the upregulated and downregulated expression of antioxidant genes affects the activity of related antioxidant enzymes in the H and F varieties and that the same genes are differentially expressed among different varieties of Chinese cabbage after stress [19].

The DEGs associated with the reactive oxygen species scavenging system in L and S varieties of Chinese cabbage under Pb stress are shown in Figure 6(2)A; 63 and 72 DEGs were significantly expressed in each of the L and S varieties after being subjected to Pb stress, (Figure 6(2)B), and as shown by Wayne plots (C) and clustered heatmaps (Figure 6(2)C,D), 49 differential genes were significantly expressed in both varieties. Among the shared genes, there were 32 GST, 1 superoxide dismutase (SOD), 12 CAT, and 4 ascorbate peroxidase (APX) genes, and the rest of the genes, except GST, were expressed in the same direction in both varieties. This indicates that the difference in the content of antioxidant enzymes between the two varieties under Pb stress was mainly due to the GST gene.

#### 2.7.2. DEGs Related to Metal Transporter Proteins

**Figure 7:**
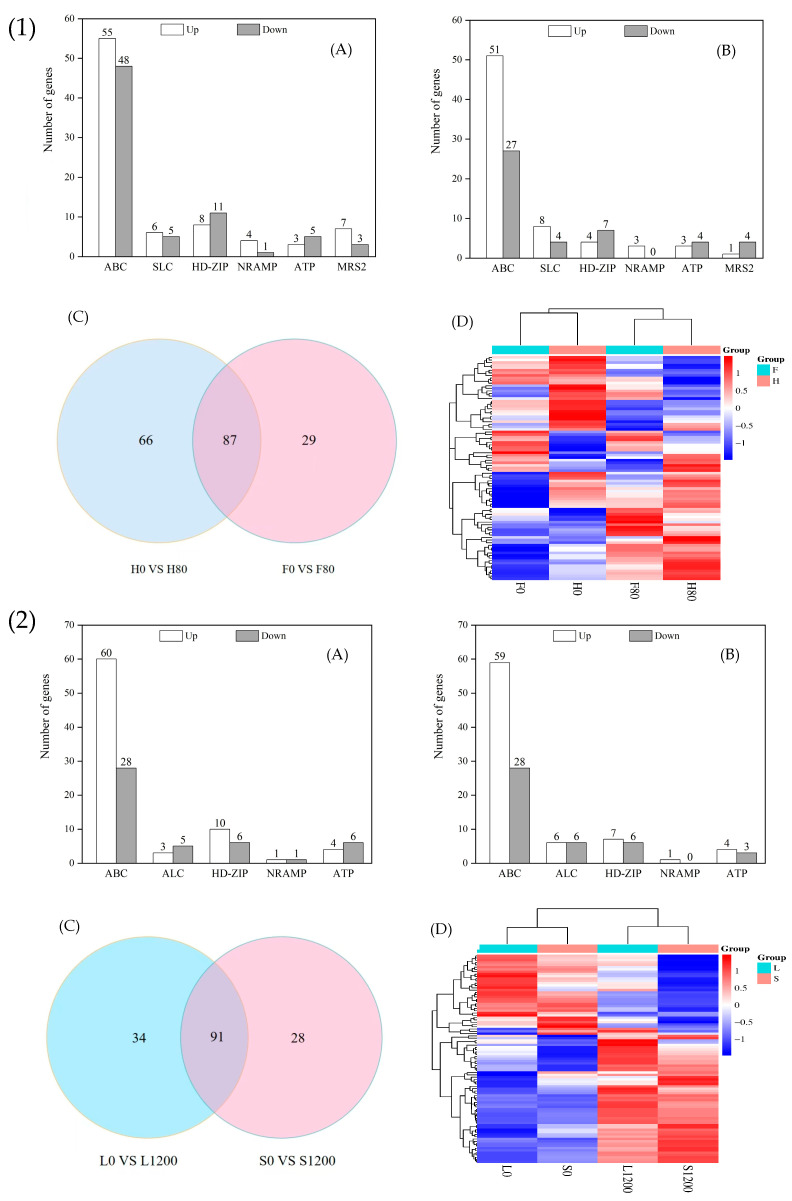
Number of significantly different genes (**A**,**B**), Wayne plots (**C**), and clustered heatmaps (**D**) related to metal transport in H and F varieties under Cd stress, and L and S varieties under Pb stress. (**1**)(**A**) is number of significantly different genes related to metal transport in H under Cd stress; (**1**)(**B**) is number of significantly different genes related to metal transport in F under Cd stress; (**1**)(**C**) is wayne plots; (**1**)(**D**) is clustered heatmaps; (**2**)(**A**) is number of significantly different genes related to metal transport in L under Pb stress; (**2**)(**B**) is number of significantly different genes related to metal transport in S under Pb stress; (**2**)(**C**) is wayne plots; (**2**)(**D**) is clustered heatmaps.

As shown in Figure 7(1)A,B, 146 and 111 DEGs were in the H and F varieties after Cd stress, of which 76 and 69 were significantly upregulated and 70 and 42 were significantly downregulated, respectively. Figure 7(2)C,D show 87 genes related to metal transport in the two varieties under Cd stress, of which 60 encoded ABC genes, 6 encoded SLC genes, 5 encoded HD-ZIP genes (among them, the genes Bra017853 (HD-ZIP22) and Bra009219 (HD-ZIP 14) were upregulated in the H variety but downregulated in the F variety); 5 encoded ATP genes (among them, Bra005531 and Bra022908 (ATPC) were upregulated in F but downregulated in H); 2 encoded MRS genes (among them, Bra008431 (MRS2-10) was upregulated in H but downregulated in F). There were genes with opposite expression directions of upregulation and downregulation in H and F, as well as genes with the same direction of expression.

As shown in Figure 7(2)A,B, 121 and 120 DEGs were in the L and S varieties after Pb stress, 75 and 60 of which were significantly upregulated and 46 and 28 genes were significantly downregulated, respectively. As shown in Figure 7(2)C,D, 91 co-expressed genes were related to metal transport in both varieties under Pb stress, of which 66 encoded ABC genes, and Bra023202 and Bra008426 (ABCG14) were upregulated for expression in L and downregulated in S, and Bra014879 (ABCC12) was upregulated for expression in S and downregulated in L.

#### 2.7.3. DEGs Related to Signal Sensing and Transduction Proteins

**Figure 8:**
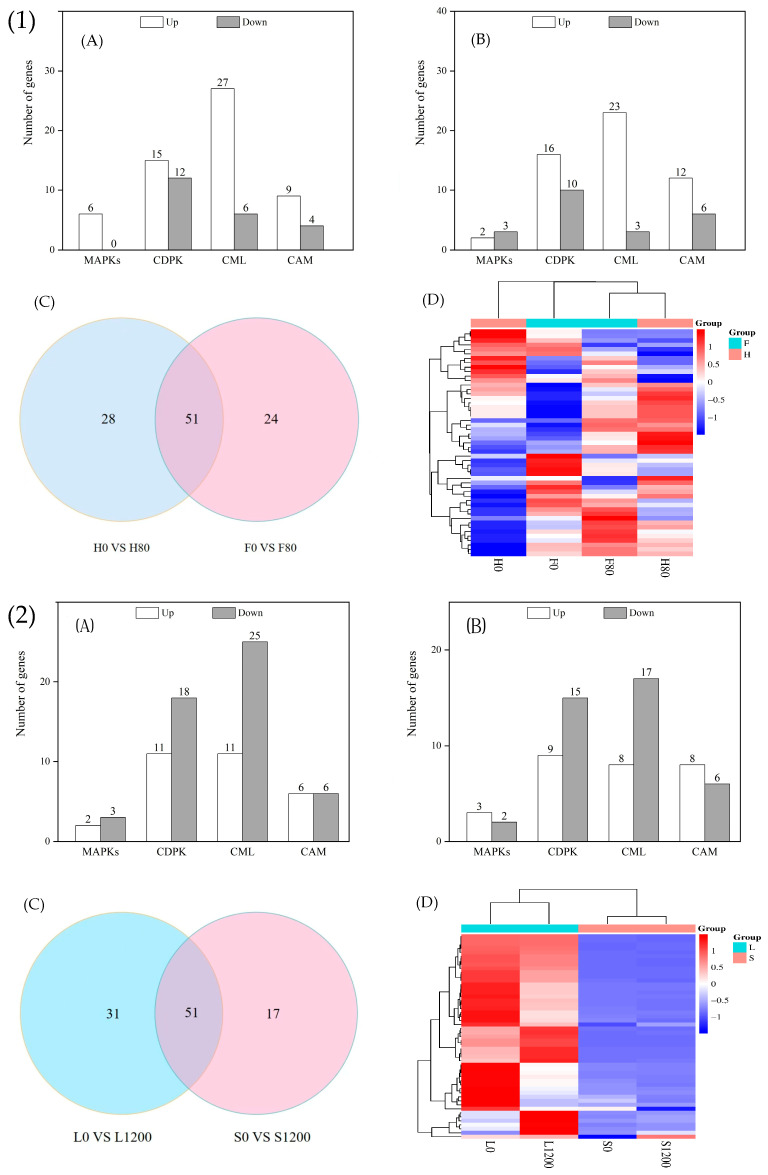
Number of significantly different genes associated with signal sensing and transduction proteins in H and F varieties under Cd stress, and L and S varieties under Pb stress. (**1**)(**A**) is number of significantly different genes associated with signal sensing and transduction proteins in H under Cd stress; (**1**)(**B**) is number of significantly different genes associated with signal sensing and transduction proteins in F under Cd stress; (**1**)(**C**) is wayne plot; (**1**)(**D**) is clustered heatmap; (**2**)(**A**) is number of significantly different genes associated with signal sensing and transduction proteins in L under Pb stress; (**2**)(**B**) is number of significantly different genes associated with signal sensing and transduction proteins in S under Pb stress; (**2**)(**C**) is wayne plot; (**2**)(**D**) is clustered heatmap.

Significant DEGs related to signal sensing and transduction proteins in the H and F varieties after Cd stress are shown in Figure 8(1)A,B; 79 and 75 genes were significantly expressed in the H and F varieties after Cd treatment, respectively. There were 6 genes encoding mitogen-activated protein kinases (MAPKs), 27 genes encoding calcium-dependent protein kinase (CDPK), 33 genes encoding calcium-binding protein (CML), and 1 gene encoding calcium-dependent protein kinase (CDPK). The binding protein CML and 13 genes encoding calmodulin (CAM) were significantly expressed in the H variety, whereas 5 genes encoding MAPKs, 26 genes encoding CDPK, 26 genes encoding CML, and 18 genes encoding CAM were significantly expressed in the F variety. As shown in Figure 8(1)C,D, there were 51 significantly expressed genes related to signal sensing and transduction proteins in the H and F varieties after Cd stress, including 4 genes encoding MAPKs; 15 genes encoding CDPKs (among these, the genes Bra002099 (CDPK16), Bra015896 (CDPK30), and Bra003287 (CDPK32)) were upregulated in H but downregulated in F; the gene Bra005824 (CDPK1) was upregulated in the F variety and downregulated in the H variety; 20 genes encoding CMLs and 4 genes encoding CAMs were also downregulated. 

As shown in Figure 8(2)A,B, there were 82 and 68 DEGs in the L and S varieties after Pb stress, respectively, with 30 and 28 genes significantly upregulated and 52 and 40 genes significantly downregulated, respectively. As shown in Figure 8(2)C,D, 51 significantly expressed genes related to signal sensing and transduction proteins were seen in the L and S varieties after Pb stress, including 3 genes encoding MAPKs, 21 genes encoding CML, 11 genes encoding CAM, and 17 genes encoding CDPK (among these, the Bra009697 (CDPK12)) gene was upregulated in L but downregulated in S; the Bra037181 (CDPK28) gene was upregulated in S and downregulated in L.

### 2.8. Transcription Factor Analysis of DEGs

**Figure 9:**
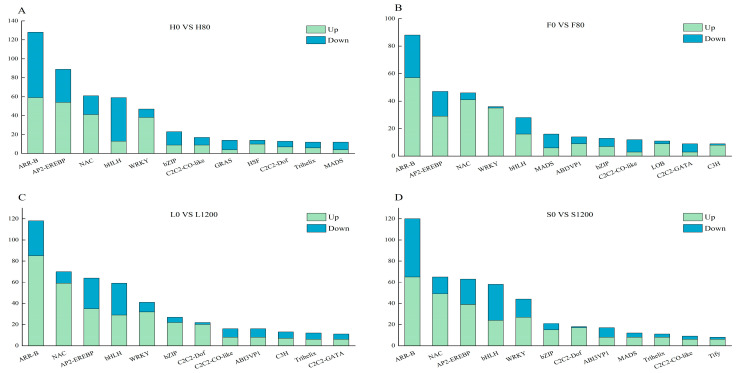
Number of transcription factors for DEGs. (**A**) is number of transcription factors for DEGs in H; (**B**) is number of transcription factors for DEGs in F; (**C**) is number of transcription factors for DEGs in L; (**D**) is number of transcription factors for DEGs in S.

Many transcription factors are involved in abiotic and biotic stress responses and play important regulatory roles in plant growth, development, and stress responses. In the two genotypes of Chinese cabbage under Cd and Pb stress, 881 and 858 genes were identified to be differentially expressed and were classified into 47 and 46 transcription factor families, respectively. The top 12 transcription factor families with the most DEGs in different treatments were selected to draw the histograms (Figure 9), and the expression levels of the different families in different genotypes of Chinese cabbage under the same Cd and Pb treatment levels differed. ARR-B, AP2-EREBP, NAC, bHLH, and WRKY were the top five families with the highest number of DEGs in all four varieties.

### 2.9. RT-qPCR Validation

**Figure 10:**
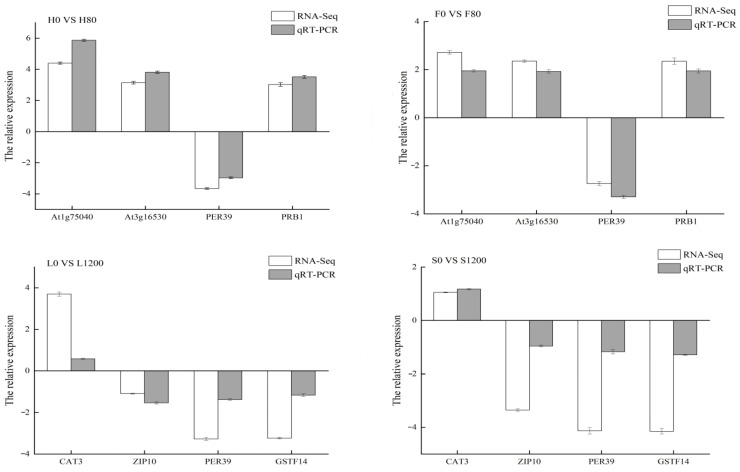
Results of RT-qPCR validation of differential genes.

To verify the validity and reliability of the RNA-Seq sequencing results, four DEGs were randomly selected from each of the Cd and Pb treatments for RT-qPCR to verify their expression levels. As shown in Figure 10, Atlg75040, At3g16530, and PRB1 were upregulated in H and F varieties under Cd stress, with RNA-Seq differential multiplicity ranging from 3.03 to 4.39, and RT-qPCR differential multiplicity of expression ranging from 3.52 to 5.87; the PER39 gene was downregulated in both varieties, with RNA-Seq and RT-qPCR multiplicity of expression ranging from −3.66 to −2.97. Under Pb stress, the CAT3 gene was upregulated in L and S varieties, with RNA-Seq and RT-qPCR multiplicity of expression ranging from 1.05 to 1.17; ZIP10, PER39, and GSTF14 genes were downregulated in both varieties, with RNA-Seq multiplicity of expression ranging from −4.15 to −3.35, and ZIP10 and GSTF14 genes were downregulated. The RNA-Seq differential folds ranged from −4.15 to −3.35, and the RT-qPCR differential folds ranged from −1.28 to −0.96. Although there were some differences between the RNA-Seq and RT-qPCR differential expression fold changes in the above genes, the RT-qPCR results accurately verified the upregulated and downregulated expression characteristics of each DEG. Therefore, the RNA-Seq results of this study are reliable.

## 3. Discussion

### 3.1. GO and KEGG Enrichment Analysis of DEGs

In GO enrichment analysis, most of the DEGs under Cd stress are enriched in the fixed components of membranes and membrane-intrinsic components in cellular fractions [20]. And the low-accumulation variety H under cadmium stress showed a high enrichment of hydrolytic O-glycoside compounds, transfer activity, hexosyl transfer, transfer activity, transfer glycosylation, and carboxylesterase activity, indicating that these DEGs respond to stress by actively transporting Cd to reduce its absorption by transporters when plants are subjected to trace heavy metal stress. The DEGs of the high-accumulation variety F were primarily enriched in anion transmembrane transporter activity, inorganic anion transmembrane transporter activity, and substrate-specific transmembrane transporter activity. When exceeding the critical threshold, stress responses occur, transporting Cd to the underground parts for accumulation, thereby slowing plant growth [21,22,23]. Yang Yining et al. [24] demonstrated that Ivy vulgaris produced a stress response under low and medium concentrations of Pb stress and showed a stronger response to all stimuli. Zhang Siqi [25] found that plants show a series of oxidative stress responses due to Pb stress, and the results of the present study were consistent with them, which further verified the reliability of the research hypotheses. Although the large enrichment of differential genes in stress-response-related entries in BPs is for defense against the hazards caused by Pb stress, both L and S varieties have a strong self-regulatory ability against Pb stress. 

KEGG enrichment analysis showed that a large number of genes were annotated for secondary metabolism biosynthesis, phenylpropanoid biosynthesis, and other metabolic pathways in different Chinese cabbage varieties under Cd and Pb stress. Metabolism and secondary metabolism are the result of plant interactions with the environment during long-term evolution and are the main regulatory mechanisms that improve plant stress tolerance. Dong Chunlan [26] found that metabolic pathways, secondary metabolism biosynthesis, ribosomal pathways, and other pathways play important regulatory roles in Fengdan’s adaptation to Cu stress, which is consistent with the results of Daijing Zhang [27]. The phenylpropane metabolite pathway mainly includes lignin and proanthocyanidin-specific synthesis pathways, which can scavenge reactive oxygen radicals, slowing membrane peroxidation [28]. Lignin enhances the strength of physical barriers and utilizes its chemical structure to effectively “restrict” or “isolate” most heavy metal ions within the cell walls of the root epidermis, cortex, and even inner cortex of plants. This compartmentalization is a key component of plants’ detoxification strategies. Root systems can fix heavy metals in their external tissues, preventing their long-distance transport to aboveground parts. This is of great significance for crops, as it reduces the accumulation of heavy metals in edible parts, ensuring food safety [29].

### 3.2. Analysis of Cd and Pb Stress Defense- and Detoxification-Related Genes

Plants subjected to heavy metal stress will produce a series of defense- and detoxification related mechanisms to avoid or mitigate the damage, and these regulatory mechanisms mainly include reactive oxygen species scavenging and antioxidant enzymes, metal transporter proteins, and signal sensing and transduction proteins. Together, SOD, CAT, POD, APX, and GST constitute an antioxidant enzyme system that can directly or indirectly scavenge reactive oxygen species in the body, which is one of the major defense mechanisms against oxidative stress [30]. In this study, genes related to antioxidant enzymes under Cd stress were co-expressed in 54 differential genes in H and F varieties; genes related to antioxidant enzymes under Pb stress were co-expressed in 49 differential genes in L and S varieties; and genes significantly upregulated the expression of SOD, APX, CAT, TRX, and GST genes, which suggests that increasing antioxidant enzyme levels is one of the important mechanisms of Cd tolerance in Caillea, deepening the understanding of the mechanism of heavy metal tolerance in plants and providing an important theoretical basis for the cultivation of resistant crop varieties.

ABC transporter proteins are involved in various important transporter processes in the plant body and can transport metal ions or other substances to vesicles to increase plant tolerance [31]. In the present study, 60 genes encoding ABC transporter proteins were identified as activated under Cd stress, of which 39 genes encoding ABC were upregulated and expressed in H varieties and 36 genes encoding ABC were upregulated and expressed in F varieties. A total of 66 genes encoding ABC were identified as activated under Pb stress, of which 47 genes encoding ABC were upregulated and expressed in L varieties and 44 genes encoding ABC were upregulated and expressed in S varieties. Similar results were found by Dong Pengcheng et al. [32], in which ABC genes such as *IbABC1, IbABCB15*, and *IbABCF4* were upregulated and expressed after Cd stress in sweet potatoes. SLC is a transmembrane protein that mediates transmembrane biofilm transport of various nutrients and metabolites, including metal ions, inorganic ions, amino acids, lipids, and sugars [33]. In this study, the SLC gene was genetically upregulated and expressed in both Cd- and Pb-stressed Chinese cabbage. The encoded calcium-transporting ATPase (ATP) can catalyze the hydrolysis of ATP on the inner side of the plasma membrane, releasing energy to maintain a low intracellular concentration of free Ca^2+^ in order to maintain normal plant growth. No yellowing or necrosis occurred.

Plants can induce cell wall activation of various adversity-responsive signaling and transduction proteins to resist heavy metal toxicity after heavy metal stress; these proteins are mainly MPKs, CDPK, CML, and CAM [34]. MAPK is an important transmitter of signals from the cell surface to the inside of the nucleus, and there are three tiers of signaling processes: mitogen-activated protein kinase (MAPK), mitogen-activated protein kinase kinase (MAPKK), and mitogen-activated protein kinase kinase kinase (MAPKKK), which regulate cell growth, development, division, stress, differentiation, and apoptosis [35]. In this study, MAPKKK17 and MAPKKK3 in the MAPK system were upregulated in H varieties, and MAPKKK17 was upregulated in L varieties, which caused different varieties of Chinese cabbage to show different characteristics after Cd and Pb stress. Chmielowska et al. [36] found that in the roots of soybean seedlings after 3 h of Cd treatment, the MAPKK2 content was significantly higher than that in the control, which was used to improve plant stress tolerance. In this study, CDPK16, CDPK30, and CDPK32 genes were upregulated and expressed in the H variety, CDPK1 was upregulated in the F variety, CDPK12 was upregulated and expressed in the L variety, and CDPK28 was upregulated and expressed in the S variety. Ca-binding proteins and calmodulin are plant-specific Ca^2+^ receptors, and when a plant is stressed, the Ca^2+^ concentration within the plant increases, leading to the activation of CML and CAM, which regulate the physiological activities of the plant [37].

### 3.3. Investigation of Transcription Factor in DEGs

Transcription factors are important regulators of plant responses to various biotic and abiotic stresses [38]. Hou Lingyan [39] et al. found that class B ARRs delay senescence and improve stress tolerance in *Arabidopsis thaliana* by promoting the transcription of cytokinin-responsive genes. In this study, 513 ARR-B genes were differentially expressed under Cd and Pb stress, indicating that ARR-B plays an important role in plant stress tolerance. NAC is a plant-specific class of transcription factor family that is involved in various plant stress responses. The N-terminal end of the NAC family of proteins contains conserved structural domains that bind to DNA and are involved in the localization of transcription factors in the nucleus. The C-terminal end of the NAC family proteins is a transcriptional regulatory region whose structure can be changed [40]. Wang Baoxiang [41] hypothesized that the transcription factor Os NAC3 is involved in the response to Cd stress in rice. In this study, we identified 202 genes in the NAC family, which accounted for 6.89% of the transcription factors. In addition, transcription factors such as AP2-EREBP, bHLH, MDAS, ABI3VIP, WRKY, and bZIP play important roles in resistance to Cd and Pb stress in *B. napus*.

## 4. Materials and Methods

### 4.1. Experimental Materials and Design

Under the hydroponic mode, four Cd and Pb high- and low-accumulating varieties of Chinese cabbage screened by the author’s team in the preliminary experiment were selected, namely, the low Cd-accumulating variety H, the high Cd-accumulating variety F, the low Pb-accumulating variety L, and the high Pb-accumulating variety S. Specific varieties are shown in Table 3. Seeds with consistent degrees of fullness were selected, sterilized with 75% alcohol for 5 min, rinsed with distilled water, placed on a water cultivation seedling sponge, and cultivated with distilled water to produce seedlings that were then cultivated with 1/2 Hoaglang nutrient solution. After the seedlings had grown to approximately 5 cm, seedlings with consistent size were selected and rinsed with distilled water and then moved into a culture box to be cultivated. During the pre-culture period, the bucket was filled with distilled water to acclimatize the plants for 24 h and then changed to 1/2 Hoaglang nutrient solution, changed to full Hoaglang nutrient solution after another 24 h, and cultured for 28 days. Two Cd treatments (0 and 80 mg/L) and two Pb treatments (0 and 1200 mg/L) were established and harvested after 10 days of incubation with Cd and Pb solutions, during which the nutrient solution was changed every 2 days. Chinese cabbage roots were obtained using a mixed sampling method after harvesting, with three biological replicates per treatment, and labeled with serial numbers, and the sample biomass and root length of Chinese cabbage were determined. The samples were quickly frozen in liquid nitrogen after sampling and then stored at −80 °C in a refrigerator for transcriptome sequencing analysis.

### 4.2. Indicators and Measurement Methods

#### 4.2.1. Determination of Cd and Pb Content in Plants

The Cd and Pb content in cabbage was determined with reference to the Determination of Cadmium in Foods of the National Standard for Food Safety (GB 5009.15-2014) and Determination of Lead in Foods of the National Standard for Food Safety (GB 5009.12-2017). Samples were decocted by microwave digestion and their Cd and Pb determined by graphite furnace atomic absorption spectrophotometry.

#### 4.2.2. Transcriptome Sequencing Data and Data Analysis

##### Sample RNA Extraction and Quality Control

RNA was extracted from the root systems of H (H0, H80) and F (F0, F80) under Cd treatment, and L (L0, L1200) and S (S0, S1200) under Pb treatment, and the total RNA of the plants was extracted using the Tiangen Polysaccharide and Polyphenol Plant Total RNA Extraction Kit (Tiangen Biochemistry Science and Technology (Beijing) Co., Beijing, China). Agarose gel electrophoresis was performed on the nucleic acid samples to check the integrity of the nucleic acid samples, whether degradation occurred, and whether there was contamination such as protein to ensure the accuracy of nucleic acid data analysis.

##### Construction of cDNA Library

Liquid nitrogen-preserved Chinese cabbage root samples were sent to Guangzhou Kidio Co., Ltd. for transcriptome sequencing of the QC-qualified libraries by Guangzhou Kidio using the Illumina HiSeq 2500 platform (Illumina Inc., San Diego, CA, USA).

##### Sequencing Data Quality Assessment and Filtering

To ensure data quality, raw data (raw reads) were filtered before information analysis to reduce the interference caused by invalid data. First, the downstream raw reads were quality-controlled using FASTP, and the data obtained after removing adapters, N% > 10%, and low-quality reads were clean reads.

##### Screening of DEGs

The FPKM value was used to calculate the gene expression level, and DESeq2 (R 4.1.2) software was used to analyze the read count data. Based on the results of the difference analysis, FDR < 0.05 and |log2FC| > 2 were used as the criteria to screen for significantly different genes.

##### GO and KEGG Enrichment Analysis of DEGs

GO enrichment analysis: The screened DEGs were compared with the GO database (http://www.geneontology.org/) (accessed on 12 June 2024) to obtain GO functional enrichment analysis and determine the significant enrichment of GO entries in DEGs.

KEGG enrichment analysis: DEGs were compared with the KEGG public database (https://www.genome.jp/kegg/) (accessed on 12 June 2024)to identify pathways that were significantly enriched in DEGs using the following formula:

(1) $$p=1-\sum_{i=0}^{m-1}\frac{\left(\frac{M}{i}\right)\left(\frac{N-M}{n-i}\right)}{\left(\frac{N}{n}\right)}$$

Note: *N* is the number of genes with pathway annotations among all genes, *n* is the number of DEGs in *N*, *M* is the number of genes annotated to a particular pathway among all genes, and m is the number of DEGs annotated to a particular pathway.

#### 4.2.3. Fluorescence RT-qPCR Validation

To validate the reliability of RNA-Seq, RT-qPCR was used to detect gene expression levels in the RNA samples. Using CYP as an internal reference gene, eight differential genes (four each in the Cd and Pb treatments) were randomly selected for RT-qPCR validation. Based on the gene sequence information, the primer design software Primer 5 was used to design the qPCR amplification primers, and the primer information is shown in Table 4. RT-qPCR was performed in a total volume of 10 μL, containing 2.5 μL of ultrapure water, 5.0 μL of ChamQ SYBR qPCR Master Mix (Novozymes, Nanjing, China), 0.25 μL of forward and reverse primer, and 2.0 μL of cDNA template. The reaction program was performed on a Pharmaceutical Analytics QuantStudio™ 5 Real-Time PCR System (Applied Biosystems, Foster City, CA, USA). Relative expression was calculated using 2^−ΔΔCT^, and three technical replicates were performed for each sample, having ensured the reliability of the data.

### 4.3. Data Statistics and Analysis

Excel 2019 was used for data statistics, SPSS Statistics 26 was used for data analysis, and one-way analysis of variance and Duncan’s test were used for the level of significant difference (*p* < 0.05). The transcriptional data were plotted using the microbiology letter platform and the Guangzhou Kidio cloud platform.

## 5. Conclusions

In this study, we analyzed the root samples of low Cd-accumulating variety H, high Cd-accumulating variety F, low Pb-accumulating variety L, and high Pb-accumulating variety S under different Cd (0 and 80 mg/L) and Pb (0 and 1200 mg/L) concentrations using transcriptome sequencing, and the molecular response mechanisms of the different genotypes of Chinese cabbage to Cd and Pb stresses were revealed. The main conclusions are as follows.

(a) Through transcriptome sequencing of the roots of different Chinese cabbage varieties under Cd and Pb treatments, GO enrichment analysis showed that Cd and Pb stress had a greater effect on the cell periphery, external encapsulation structure, cell wall, and response to external stimuli. KEGG enrichment analysis was mainly for secondary metabolite biosynthesis, metabolic pathways, and phenylpropanoid biosynthesis pathways.
(b) An analysis of the DEGs related to defense and detoxification showed that in the reactive oxygen species scavenging system, the number of upregulated genes in the four species was greater than that of the downregulated genes, and the main difference was caused by the genes encoding CAT and GST; in the metal transporter system, there were more genes that were upregulated than downregulated after heavy metal stress, and the main difference was due to the differential expression of the genes encoding ABC transporter proteins; in the signal sensing and transducer system, the genes encoding MAPKs, CDPK, CML, and CAM were all more upregulated in the H and F varieties under Cd stress, whereas those encoding CDPK and CML were more downregulated in the L and S varieties under Pb stress.
(c) Transcription factor family analysis of differentially expressed genes in Chinese cabbage roots under Cd and Pb stress revealed the presence of numerous transcription factors, such as ARR-B, AP2-EREBP, NAC, bHLH, WRKY, and bZIP, which are strongly associated with signaling regulation related to Cd and Pb tolerance.

## Figures and Tables

**Table 1:** Aboveground and underground heavy metal contents of different Chinese cabbage varieties after 10 days of plant exposure.

Variety	Aboveground		Underground	
	Cd (mg/kg)	Pb (mg/kg)	Cd (mg/kg)	Pb (mg/kg)
Lvguan (L)	9.87 ± 1.63	1.80 ± 1.71	15.96 ± 0.94	136.25 ± 1.70
Hexiekuaicai (H)	4.72 ± 2.59	9.90 ± 0.85	9.56 ± 1.39	154.78 ± 0.51
Suzhouqing (S)	10.00 ± 1.73	18.00 ± 1.00	13.21 ± 1.69	245.32 ± 0.71
Ziwei F1 (F)	10.60 ± 2.16	13.20 ± 2.71	17.69 ± 1.14	202.31 ± 1.49

Note: The positive and negative values in the table represent the standard deviation.

**Table 2:** BCE and TF of different Chinese cabbage varieties for Cd and Pb after 10 days of plant exposure.

Variety	Cd		Pb	
	TF	BCF	TF	BCF
Lvguan (L)	0.62 ± 0.08	1.88 ± 1.12	0.013 ± 0.004	0.006 ± 0.002
Hexiekuaicai (H)	0.50 ± 0.20	0.90 ± 0.23	0.064 ± 0.008	0.032 ± 0.003
Suzhouqing (S)	0.78 ± 0.19	1.90 ± 1.26	0.073 ± 0.011	0.059 ± 0.003
Ziwei F1 (F)	0.60 ± 0.11	2.02 ± 1.32	0.065 ± 0.009	0.043 ± 0.002

Note: The positive and negative values in the table represent the standard deviation.

**Table 3:** Chinese cabbage variety information.

Variety Name	Manufacturer
Hexiekuaicai (H)	Cai Yun Seed Industry Co. (Yuxi, China)
Ziwei F1 (F)	Guangxi Hengxian Zilong Seed Industry Co. (Hengxian, China)
Lvguan (L)	Cai Yun Seed Industry Co. (Yuxi, China)
Suzhouqing (S)	Hefei Hefeng Seed Industry Co. (Hefei, China)

**Table 4:** Primers used for qPCR gene expression experiments.

Gene ID	Forward Primer	Reverse Primer
CYP	GAACTTCCGTGCCCTCTG	GTCTTTGAACTTCATGCCGTA
Bra027257	ATGACCCGTGACGAAACC	GTGGGGACGACGATGAAG
Bra035235	AGCAACCAACAAGTGGAAAA	TCAAAGCCTCGGTAGCATT
Bra036984	AGTTCCTCTGAAAGCCCAAG	CCCACATCCTCACTGCGT
Bra016511	CCCCGTTTGGCCTGGAAT	CCCAGATTCTGCCGGACC
Bra035235	AGCAACCAACAAGTGGAAAA	TCAAAGCCTCGGTAGCATT
Bra012238	CCTCACTTGTGCTGATTTCC	ATGTTTGTTTTCGGGTTCG
Bra023171	CCGCTTTTCAGCCCGAGA	CGCCAAGACAAGGAGACGA
Bra037204	CGTTTAGGCGAGTCTTGTTATT	GCATACGGCGTTGTTTCA

## Data Availability

The data that support the findings of this study are available upon reasonable request from the authors.

## Abbreviations

The following abbreviations are used in this manuscript:

DEGs	Differentially expressed genes
H	Harmony Express
F	Ziwei F1
L	Green Crown
S	Suzhou Green

## Appendix A

**Table A1:** Comparison of RNA-Seq reads with reference genes under Cd stress.

Sample	Raw Data/bp	Clean Reads/bp	Q20/%	GC	Alignment Rate/%	Unique Alignment Rate/%
F0-1	38,045,654	37,847,936	97.54	47.14	85.83	83.47
F0-2	44,825,712	44,588,466	97.67	47.23	86.20	83.80
F0-3	41,441,232	41,216,564	97.40	47.16	85.68	83.31
F80-1	37,127,004	36,951,820	97.38	47.16	84.56	81.87
F80-2	37,302,860	37,145,918	97.72	47.21	84.54	81.79
F80-3	40,513,382	40,345,392	97.78	47.21	84.55	81.81
H0-1	38,133,332	37,949,190	97.70	47.19	84.47	80.24
H0-2	43,182,524	42,982,558	97.69	47.17	82.43	80.21
H0-3	36,396,510	36,198,036	97.44	47.16	84.00	81.75
H80-1	38,229,992	38,058,584	97.93	46.97	84.67	82.53
H80-2	39,930,032	39,750,840	97.79	47.00	84.53	82.37
H80-3	37,624,148	37,452,574	97.91	46.97	84.39	82.21

**Table A2:** Comparison of RNA-Seq reads with reference genes under Pb stress.

Sample	Raw Data/bp	Clean Reads/bp	Q20/%	GC	Alignment Rate/%	Unique Alignment Rate/%
S0-1	45,794,082	45,590,598	97.80	46.80	83.35	81.04
S0-2	46,843,230	46,624,614	97.40	46.84	82.14	79.89
S0-3	44,147,364	43,947,908	97.87	46.83	83.48	81.23
S1200-1	46,097,740	45,868,042	97.74	48.19	79.91	77.83
S1200-2	45,465,400	45,218,548	97.24	48.27	79.12	77.03
S1200-3	44,995,858	44,772,442	97.56	48.14	79.98	77.86
L0-1	41,901,870	41,696,094	97.62	47.28	85.93	83.43
L0-2	45,125,548	44,885,656	97.56	47.28	85.90	83.43
L0-3	41,244,174	41,048,822	97.78	47.29	86.09	83.60
L1200-1	42,175,062	41,973,480	97.69	46.97	84.54	82.43
L1200-2	42,766,174	42,532,872	97.60	46.95	84.08	81.98
L1200-3	38,192,598	38,028,248	97.87	46.97	84.37	82.26
